# Hepatitis B sero-prevalence in children under 15 years of age in South Africa using residual samples from community-based febrile rash surveillance

**DOI:** 10.1371/journal.pone.0217415

**Published:** 2019-05-31

**Authors:** Nishi Prabdial-Sing, Lillian Makhathini, Sheilagh Brigitte Smit, Morubula Jack Manamela, Nkengafac Villyen Motaze, Cheryl Cohen, Melinda Shelley Suchard

**Affiliations:** 1 Centre for Vaccines and Immunology, National Institute for Communicable Diseases (NICD), a division of the National Health Laboratory Service, Johannesburg, South Africa; 2 Department of Virology, School of Pathology, Faculty of Health Sciences, University of the Witwatersrand, Johannesburg, South Africa; 3 Division of Epidemiology and Biostatistics, Department of Global Health,Faculty of Medicine and Health Sciences, Stellenbosch University, Cape Town, South Africa; 4 Centre for Respiratory Diseases and Meningitis, National Institute for Communicable Diseases, a division of the National Health Laboratory Service, Johannesburg, South Africa; 5 School of Public Health, University of the Witwatersrand, Johannesburg, South Africa; 6 Department of Chemical Pathology, School of Pathology, Faculty of Health Sciences, University of the Witwatersrand, Johannesburg, South Africa; University of Cincinnati College of Medicine, UNITED STATES

## Abstract

**Introduction and methods:**

Hepatitis B is a vaccine preventable disease and is notifiable in South Africa. Hepatitis B vaccination was incorporated into the Expanded Programme on Immunisation in South Africa in 1995. We used a convenience sample from community-based febrile rash surveillance in 2013 to estimate hepatitis B sero-prevalence. Of samples serologically negative for acute measles infection, 450 samples spanning nine provinces of South Africa were tested for hepatitis B surface antigen (HBsAg), hepatitis B surface antibody (anti-HBs) and hepatitis B core antibody (anti-HBc).

**Results:**

Two children (2/450; 0.4%) tested positive for HBsAg. Three hundred and three children (67.3%) had evidence of vaccine induced immunity. Vaccine induced immunity was present in 80.2% of 1–5 year olds, but only 60.3% of 10–14 year olds. Natural immunity, indicating exposure to circulating hepatitis B, was present in 13/450 (2.9%) children.

**Conclusion:**

Chronic hepatitis B in South African has decreased in prevalence from highly endemic levels prior to vaccine introduction to approximately 0.4% in this sample, demonstrating impact of a successful vaccination programme 18 years after introduction. Decreased vaccine-induced immunity with increasing age may reflect waning antibody titres over time.

## Introduction

Hepatitis B is a vaccine preventable disease and is notifiable in South Africa. There is no active national surveillance for hepatitis B. We estimated hepatitis B seroprevalence in South African children using residual sera from febrile rash surveillance.

Prior to vaccine introduction, South Africa was considered a country of high hepatitis B endemicity, defined as ≥8% prevalence for hepatitis B surface antigen (HBsAg) in the general population[1]. Rates of 5–16% for hepatitis B surface antigen were seen in rural black men, with lower rates in females, urban populations and other ethnic groups [2–5]. In the late 1980s in Kwazulu-Natal province, rates of 18.5% in rural and 10% in urban black children were reported, with rates of 2.5% in urban children under 6 years [6], while rates of 1% were reported in urban children 1–19 years of age in Soweto, Gauteng province [7]. Areas of the Eastern Cape in the 1990s had prevalence rates of 10% in children less than 6 years old [8]. Paediatric hepatitis B in South Africa in the pre-vaccine era was reported to be mostly due to horizontal transmission in childhood rather than vertical transmission from mother to child. Modes of horizontal transmission are not well defined but may result from exposure amongst children to infectious saliva or open sores amongst other postulated mechanisms [3, 6, 9–12].

Hepatitis B vaccination was incorporated into the routine Expanded Programme on Immunisation in South Africa in 1995, administered at 6, 10 and 14 weeks of age. An 18-month booster dose was later added in 2015, as part of a hexavalent preparation [13]. The World Health Organisation and United Nations International Children’s Emergency Fund (UNICEF) estimates of third dose hepatitis B vaccine coverage range from 66–85% from 2006 to 2017 [14]. A birth dose was not part of the South African immunisation schedule during this period. Since universal hepatitis B vaccine introduction into infant schedules, prevalence of chronic hepatitis B infection, measured by presence of hepatitis B surface antigen has decreased globally [15]. High HIV prevalence rates in South Africa may, however, influence rates of vertical transmission to infants or chronic carriage amongst children[16, 17].

In 2012, HIV prevalence in South Africa was estimated at 12.2% overall, with prevalence of 2.4% in children 2–14 years [18]. In studies published since 2000, HBsAg prevalence in South African adults living with HIV has been reported as 0.4–9.4% in HIV clinics [19–28] and up to 22.9% using hospital-based residual sera [29–31]. In antenatal clinics, rates of HBsAg in pregnant women ranged from 0.4–5.8% in HIV-uninfected women and 2.1–7.4% in HIV-infected women [32–35]. Regarding influence of HIV on hepatitis B vertical transmission from mother to child, Chotun et al found 0.3% of infants born to HIV-infected women positive for HBsAg, dropping to 0.2% on follow up [16]. Hoffman et al found 0.5% of infants born to HIV infected mothers positive for HBsAg [17]. Regarding hepatitis B seroprevalence in children, Jooste et al screened South African HIV-infected children and adolescents and found 0.8% positive for HBsAg [36]. In outpatient clinics, Simani et al found HBsAg prevalence of 0.4% in children under two years old (with higher prevalence of 2.7% in HIV-infected children) [37].

Community-based rash surveillance is used as a detection system for possible measles infection. Serum samples of children who have rash and fever together with cough, coryza or conjunctivitis are sent to the National Institute for Communicable Disease for measles serology. Approximately 7000 samples are tested annually, of which usually less than 50 are positive for measles IgM. The remaining samples are from children with acute rash illness other than measles. Rash in children has many aetiologies including viral (measles, rubella, ECHO, coxsackie, parvovirus B19, herpesvirus 6 and others), bacterial and rickettsial infections and other non-infectious causes. Rash therefore may affect any sector of the population including multiple ethnic, socioeconomic and demographic groups.

In this study, hepatitis B infection, exposure and protection was measured in children less than 15 years of age. Residual surveillance samples were used as a proxy for children in the general community.

## Methods

### Sample selection

Of 7107 rash samples collected in 2013, 450 samples were sequentially selected from children less than 15 years of age that tested negative for measles and rubella IgM. Samples with insufficient serum volume (<600μl) were excluded. Hepatitis B surface antigen (HBsAg), hepatitis B surface antibody (anti-HBs) and hepatitis B core antibody (anti-HBc) were tested. HBsAg positive samples were further tested for e antigen (HBeAg), antibodies to e antigen (anti-HBe), viral load and genotyping.

### Laboratory tests

HBsAg and anti-HBc were run on the Architect instrument (Abbott, Germany) and values equal to or greater than 1.00 Sample/Cutoff (S/CO) were considered positive. Anti-HBs titres (Architect, Abbott, Germany) were reported as milli-international units per ml (mIU/ml) according to WHO international reference standard (Hollinger and Dreesman, 1986). Samples equal to or greater than 10mIU/ml were considered positive for anti-HBs. Samples positive for HBsAg were tested for HBeAg and anti-HBe (Architect, Abbot, Germany) with cutoff at 1.00 S/CO.

Hepatitis B viral load was performed on HBsAg positive samples using the COBAS AmpliPrep/COBAS TaqMan HBV Test, v2.0, quantitative assay (Roche Molecular Systems, New Jersey, USA). Sequencing was performed on the polymerase region (nucleotides 2624–1240), overlapping the complete S region (nucleotides 2848–2835) using BigDye Terminator v3.0 Cycle Sequencing Ready Reaction Kit (Applied Biosystems, Foster City, CA, USA) on the ABI 3130XL Genetic Analyzer (Applied Biosystems) [38]. Neighbour-joining phylogenetic analysis with a bootstrap of 1000 replicates was conducted using Molecular Evolutionary Genetics Analysis (MEGA 5)[39].

### Data analysis

Results are reported as indicative of chronic infection (HBsAg positive), vaccine induced immunity (HBsAg negative, anti-HBs positive,anti-HBc negative) or natural exposure (HBsAg negative, anti-HBc positive).

### Sample size

Sample size calculation was conducted using Open-Epi software. Based on an anticipated 2% prevalence of HBsAg positive samples, with a 99% confidence level, confidence limits of +/- 2% and a design effect of 1, a minimum sample size of 350 samples was needed. The study was not powered to estimate hepatitis B prevalence stratified by age group or by province.

### Ethics

This study was approved by the University of Witwatersrand Human Research Ethics committee (clearance certificate M140857). Subjects were not required to give informed consent as samples had been collected during routine febrile rash surveillance and were anonymized for this study.

## Results

Of 450 samples selected, 47% were females and 53% were males, with a median age of 5 years. Ages of included children were 11 aged 0–11 months, 217 aged 1–5 years, 174 aged 6–10 years and 48 aged 11–14 years (2.4%, 48%, 38.7% and 10.7% respectively). Provincial distribution of samples included Mpumalanga 14% (n = 65) Eastern Cape 13% (n = 59), North West Province 13% (n = 58), Northern Cape 12% (n = 55), KwaZulu Natal 11% (n = 51), Free State 10% (n = 43), Limpopo 10% (n = 47), Gauteng 9% (n = 42) and Western Cape 7% (n = 30).

HBsAg was positive in two children, 0.4% (2/450) of the sample, a 2 year-old from Ehlanzeni in Mpumalanga province (HBeAg positive, viral load 4.6log_10_copies/ml) and a 12-year-old from Ethekwini Metro in KwaZulu-Natal (HBeAg negative, anti-HBe positive, viral load 2.6 log_10_copies/ml). Both children were positive for anti-HBc and both samples were genotype A1.

Vaccine induced immunity (individuals positive for anti-HBs with negative anti-HBc [40]) was high in children under 1 year (81.8%) and 1–5 years (80.2%) but lower in older age groups (60.3% in 6–10 year olds and 31.3% in 11–15 year olds (Table 1). Overall, 2.9% of children (13/450) had evidence of natural exposure (anti-HBc) to hepatitis B, implying they had been exposed to circulating HBV at some time. Of the 450 samples tested, 132 (29.3%) had no serological markers positive, indicative of either no exposure to HBV, no exposure to hepatitis B vaccine, or perhaps prior vaccine induced immunity which may have waned with time.

**Table 1:** Hepatitis B serology in residual sera from patients with febrile rash illness, by age group.

* *	Age groups (n)									
	0-11months		1-5years		6-10years		11-14years		Total	
	n	%	n	%	n	%	n	%	n	%
Infected (HBsAg+)	0	0.0	1	0.5	0	0.0	1	2.1	**2**	**0.4**
Vaccine Immunity (anti-HBs+ and anti-HBc-)	9	81.8	174	80.2	105	60.3	15	31.3	**303**	**67.3**
Natural exposure (anti-HBc+)	1	9.1	5	2.3	6	3.4	1	2.1	**13**	**2.9**
No evidence of natural exposure or vaccine immunity (HBsAg-, anti-HBs- and anti-HBc-)	1	9.1	37	17.1	63	36.2	31	64.6	**132**	**29.3**
**Total**	**11**	**100.0**	**217**	**100.0**	**174**	**100.0**	**48**	**100.0**	**450**	**100.0**

HBsAg hepatitis B surface antigen; anti-HBs Hepatitis B surface antibody; anti-HBc Hepatitis B core antibody; + positive

- negative; n number. Percentages shown in darker shade with totals reflecting column totals.

## Discussion

Our finding of 0.4% prevalence of hepatitis B infection in children under 15 years of age adds an estimate of burden of infection in the post vaccine era. While other studies have focused on targeted clinics (antenatal, HIV, immunisation, outpatient clinics) or used hospital-based residual sera, we have studied a community-based population. We found two cases positive for HBsAg, which may reflect either horizontally or vertically acquired infections.

Vaccine induced immunity was highest in infants and young children, decreasing in older age groups.

Age dependent decreases in rates of vaccine induced immunity could imply absence of vaccination, biological non-response to vaccine or waning of immunity. Reduction in immunity as age increases has been previously reported. [41–45]. Protection against infection may endure despite waning of anti-HBs titres, as immunologic memory produces anamnestic responses to boosters [45, 46]. Anti-HBs prevalence is therefore not a reliable indicator of community vaccination coverage or immunity to hepatitis B infection and should be interpreted with caution.

HIV infection was not tested in our samples, due to ethical sensitivities. We therefore cannot analyse whether carriage rates of hepatitis B in children differ in those who are HIV infected or exposed from those who are HIV uninfected. Occult hepatitis B infection was not investigated. Occult hepatitis B, usually considered as detectable hepatitis B deoxyribonucleic acid (DNA) but undetectable HBsAg, has previously been reported in both HIV negative and HIV positive adults [20, 29, 30, 34, 35, 47] and HIV exposed infants [16, 17, 48] in South Africa. In patients with occult hepatitis B, antibody against Hepatitis B core antigen is often detected. The clinical implications of occult hepatitis B infection for patient prognosis thus remain obscure [49]. HBsAg remains the most frequently used test for prevalence in most epidemiological surveys and allows comparisons between countries and over different time periods.

Relatively few samples were included from patients under one year of age, due to insufficient residual serum volume from most infant samples. Infant samples can give information regarding suspected vertical transmission, although horizontal transmission may also occur in the first year of life. Infant sampling may also be confounded by maternal antibody levels. Babies born to HBsAg-positive mothers who do not become infected may be positive for anti-HBc for up to 24 months after birth from passively acquired maternal antibody [49].

We are unable to comment on rural versus urban origin of individuals included due to limited information. Prior to introduction of universal hepatitis B vaccination in South Africa, rates in urban areas were lower than in rural areas. Our samples may be biased to children originating from urban areas, who would be more likely to seek health care during febrile rash illness. Rural and urban districts actively participate in febrile rash surveillance, however, and are required to meet similar targets for surveillance adequacy. The private sector may be under-represented as surveillance officers are employed by national and provincial departments of health rather than private organizations. Surveillance officers are, however, tasked with active surveillance in both public and private institutions. The study was not powered to analyse whether differences existed in rates of HBsAg carriage per province. Increasing the sample size to include representative numbers of samples from each province could be performed in future to give geographic estimates of disease prevalence.

Previous studies on hepatitis B in the post- vaccination era have largely been conducted in localized health-care facilities or from tertiary referral centres, with the majority of patients drawn from one or two provinces. A strength of our study is the national distribution of samples included. Despite the non-measles febrile rash illness of our participants, they are unlikely to be drawn from particularly high or low risk groups for vaccination completion as viral rash illness is not vaccine preventable and patients with rash are therefore a “biologically arbitrary” selection. Since febrile rash surveillance is performed in a similar fashion by most WHO member states, this study design represents a convenience sample that could be replicated in other countries in order to inform regional prevalence and immunity figures. Representivity of rash surveillance samples may be skewed in countries with routine rubella immunisation, where febrile rash samples may be biased towards unvaccinated children. Rubella immunisation is not currently part of the South African routine immunisation schedule.

In conclusion, we report HBsAg prevalence of 0.4% in South African children from a period in which the Expanded Programme on Immunisation schedule included three primary doses but no booster or birth dose. While studies with active community-based random sampling are the gold standard for seroprevalence studies, residual samples from febrile rash surveillance are a low-cost proxy that can be repeated annually and can offer insights regarding disease trends over time. Febrile rash surveillance samples could also be used to study seroprevalence of other diseases.

## Supporting information

S1 Supporting Dataset (XLSX)
